# Supplementary material for: Application of 1H-NMR Metabolomics for the Discovery of Blood Plasma Biomarkers of a Mediterranean Diet
Source: Metabolites. 2019 Sep 27;9(10):201. doi: 10.3390/metabo9100201 (PMC6836148; doi:10.3390/metabo9100201)
Supplement: Supplementary file 1 [file metabolites-09-00201-s001.zip › metabolites-572354-SI.pdf]

Table S1. Correlations of the top 5 metabolites correlations with MDS and each of the food groups assessed in the 14-item MD score.

Food group	Citric Acid			Mannose			Pyruvic Acid			*Myo*-Inositol			Betaine		
	r	p	Q	r	p	Q	r	p	Q	r	p	Q	r	p	Q
**MD score**	**0.28****	0.001	0.26	**-0.20***	0.02	0.63	**-0.02****	0.008	0.56	**-0.18***	0.03	0.63	**0.19***	0.02	0.63
**Fruit (Fr)**	0.15	0.07	0.80	**-027****	0.002	0.31	**-0.23****	0.007	0.56	-0.16	0.06	0.88	0.05	0.55	0.91
**Fruit Juice (J)**	**0.18***	0.03	0.70	-0.10	0.23	0.90	-0.05	0.56	0.91	-0.01	0.85	0.98	**0.19***	0.03	0.64
**Fruit and Fruit Juice (FJ)**	**0.22***	0.02	0.63	**-0.27****	0.002	0.31	**-0.19***	0.02	0.63	-0.11	0.18	0.73	0.15	0.07	0.81
**Vegetables (V)**	0.02	0.83	0.97	-0.02	0.79	0.97	-0.06	0.50	0.91	-0.12	0.15	0.88	0.06	0.44	0.91
**Fr & J & V combined (FJV)**	**0.20***	0.02	0.63	**-0.23****	0.007	0.31	-0.15	0.07	0.80	-0.16	0.06	0.71	**0.20***	0.02	0.63
**Red meat (RM)**	-0.06	0.48	0.91	0.09	0.25	0.90	0.13	0.11	0.86	0.10	0.24	0.90	-0.01	0.90	0.99
**Chicken and Turkey (CT)**	0.04	0.06	0.93	-0.14	0.10	0.86	-0.11	0.19	0.90	0.06	0.95	0.99	0.02	0.79	0.97
**Fish (F)**	0.13	0.12	0.86	-0.02	0.78	0.97	**-0.32****	<.0001	0.18	-0.14	0.10	0.83	0.12	0.14	0.88
**Nuts (N)**	0.06	0.52	0.91	0.02	0.84	0.98	-0.13	0.13	0.88	-0.15	0.09	0.83	0.04	0.68	0.95
**Processed meat (PM)**	-0.02	0.79	0.97	-0.004	0.96	0.99	**0.22****	**0**.009	0.59	**0.18***	**0.03**	0.63	-0.02	0.85	0.98
**Legumes**	0.11	0.19	0.90	-0.06	0.46	0.91	0.02	0.77	0.97	0.02	0.81	0.97	0.10	0.13	0.86
**Alcohol (A)**	0.02	0.82	0.97	0.09	0.27	0.90	-0.09	0.28	0.90	0.02	0.82	0.97	0.07	0.41	0.91
**Olive spreads (OS)**	0.07	0.40	0.90	-0.12	0.14	0.88	**-0.19***	0.02	0.63	**-.021***	0.01	0.63	0.05	0.54	0.91
**Olive / Rapeseed oil (OR)**	0.11	0.21	0.90	-0.07	0.35	0.90	-0.09	0.31	0.90	-0.05	0.54	0.91	0.09	0.29	0.90

**Sweet foods (S)**	**-0.19***	0.03	0.63	-0.03	0.64	0.93	-0.15	0.06	0.73	0.11	0.22	0.90	-0.11	0.21	0.90
**Sweet foods & drinks (SD) (SD)**	**-0.19***	0.03	0.63	-0.13	0.12	0.86	-.005	0.58	0.91	0.03	0.69	0.95	-0.08	0.33	0.90
**Whole grain cereals (C)**	0.03	0.74	0.96	**-0.21***	0.015	0.63	-0.09	0.30	0.90	-0.04	0.67	0.95	0.05	0.53	0.91

Spearman's rank correlation coefficients (r) and false discovery rates (Q) were obtained for the top five discriminant metabolites to indicate those significantly correlated with the 14-item MD score. Individual food group correlations coefficients and its false discovery rates were also identified for citric acid: (J, FJ, FJV, S and SD); mannose: (Fr, FJ, FJV, and C); pyruvic acid: (Fr, FJ, F, PM and OS) and *Myo*-inositol (PM and OS) and Betaine: (J and FJV). Significance is indicated as $*P < 0.05$, $**P < 0.01$.

Commented [SM1]: I was just wondering if it would be possible to reduce the font size of the number from 10 to 9 to avoid altering the format of the table

Table S2. Baseline characteristics of study participants.

Variable	Low MDS	High MDS	P-value
**Age (years) Mean (SD)**	56.13 (9.37)	60.54 (7.88)	0.12
**Blood Pressure: Systolic (mmHg) Mean (SD)**	122.02 (15.98)	132.84 (17.74)	0.04*
**Blood Pressure: Diastolic (mmHg) Mean (SD)**	66.72 (9.22)	73.57 (8.29)	0.02*
**BMI (kg/m²) Mean (SD)**	30.23 (5.14)	29.77 (6.81)	0.79
**Smokers %**	26.66	0	
**Sex: Male %**	77.78	92.31	
**Sex: Female %**	22.22	7.69	

Patients general characteristics measured at baseline (n=58). Mean age was 56.13 for the low MDS group and 60.54 for the high MDS group. Mean BMI was 30.23 kg/m² for the low MDS group and 29.77 for the high MDS with no significant differences in BMI between the two groups. Mean BMI of 30.23 and 29.77 indicate that most patients were obese and overweight respectively.

Table S3. Assigned MDS scores listed by group.

	Low MDS (n = 64)					High MDS Group (n = 73)					
**MDS**	**0**	**1**	**2**	**3**	**4**	**5**	**6**	**7**	**8**	**9**	**10**
Samples (n)	n=3	n=9	n=15	n=15	n=22	n=18	n=21	n=10	n=16	n=6	n=2

Table shows the number of individuals assigned each Mediterranean diet score (MDS). Groups were divided at the median. Group 1 (0-4): Low MDS; Group 2 (5-10): High MDS.

Table S4. Performance of multivariate modelling of metabolomic data.

Measure	1 component	2 components	3 components
Accuracy	0.546	0.646	0.694
R2	0.219	0.319	0.389
Q2	-0.006	0.055	0.039

Cross validation of the PLS-DA multivariate model as performed using Metaboanalyst 4.0.

Table S5. Food group-to-Food group correlations within the study cohort.

Food group		Fruit	Fruit Juice	Legumes	Red meat	Proc. meat	Fish	Nuts	Alcohol	Sweets & Carbonated Drinks	Olive spread	Olive/Rapeseed Oil	Chicken & Turkey	Cereals (WG)	Sweets
**Vegetables**	**r**	**0.22***	0.04	-0.11	0.01	**-0.37*** **	**0.19***	0.**21***	0.16	**-0.21***	0.08	0.**17***	-0.04	-0.07	**-0.18***
	***p*-value**	0.01	0.67	0.19	0.93	7.5E-06	0.027	.013	0.06	0.01	0.37	0.048	0.60	0.43	0.03
**Fruit**	**r**		**0.19***	0.08	-0.12	**-0.36*** **	**0.46****	0.16	-0.12	**-0.38****	**0.33****	**0.28****	**0.24*** **	**.18***	**-0.36****
	***p*-value**		0.02	0.35	0.15	1.3E-5	1.9E-08	0.06	0.14	4.5E-06	7.4E-5	0.001	0.05	0.03	1.4 E-5
**Fruit juice**	**r**			**0.24****	0-.05	-0.07	**0.18***	**0.23****	**-0.19***	**0.01**	**0.25****	0.08	**0.19***	0.13	-0.07
	***p*-value**			0.005	0.51	0.38	0.04	0.006	0.03	0.91	0.003	0.35	0.026	0.13	0.42
**Legumes**	**r**				**0.23****	**0.23****	0.04	0.15	0.16	-0.01	-0.01	0.12	0.03	0.17	-0.10
	***p*-value**				0.006	0.006	0.62	0.08	0.06	0.88	0.88	0.14	0.73	0.05	0.24
**Red meat**	**r**					**0.26****	**-0.19***	**-0.20***	**0.20***	0.08	-0.09	0.15	-0.10	0.07	0.04
	***p*-value**					0.003	0.030	0.019	0.02	0.30	0.31	0.07	0.22	0.41	0.62
**Processed meat**	**r**						**-0.34****	**-0.26****	-0.03	**0.18***	-0.09	-0.07	0.02	0.13	0.12
	***p*-value**						4.8x10^{-5}	0.002	0.76	0.037	0.26	0.44	0.86	0.13	0.15
**Fish**	**r**							0.12	0.05	**-0.26****	**0.27****	**0.17***	-0.04	0.12	**-0.14***
	***p*-value**							0.15	0.54	0.002	0.002	0.048	0.67	0.15	**0.036**
**Nuts**	**r**								0.04	-0.01	0.11	-0.01	0.13	0.03	-0.05
	***p*-value**								0.43	0.89	0.18	0.92	0.13	0.71	0.53

**Alcohol**	**r**	-0.12	**-0.20***	0.12	**-0.20***	-0.02	-0.14
	***p*-value**	0.16	**0.019**	0.14	**0.023**	0.81	0.10
**Sweets & Drinks**	**r**		-0.13	-0.12	-0.02	0.01	**0.84****
	***p*-value**		0.11	0.17	0.78	0.88	3.94E-38
**Olive spread**	**r**			**0.18*****	0.16	**0.22*****	-0.13
	***p*-value**			0.003	0.06	0.01	0.11

Spearman's rank correlation coefficients were obtained with significance is indicated as **P* < 0.05 and ***P* < 0.01

Table S6. Correlations of all 59 metabolites against each food group scored within the MDS.

Metabolites		Fruit incl. juice	Fruit excl. juice	Fruit Juice	Vegetables	Fruit, Fruit Juice & Vegetables	Legumes	Olive/ rapeseed oil	Olive spreads	Nuts	Fish	Red meat	Processed meat	Chicken & turkey	Cereals	Alcohol	Sweets & Drinks	Sweets
**1-Methylhistidine**	r	0.060	-0.029	0.118	-0.015	0.083	0.098	0.059	0.096	0.054	0.009	0.028	0.137	0.056	0.108	-0.063	0.040	0.086
	p	0.488	0.739	0.170	0.860	0.339	0.256	0.498	0.268	0.531	0.918	0.746	0.113	0.517	0.210	0.468	0.643	0.318
**2-Hydroxybutyric acid**	r	-0.075	-0.015	-0.063	-0.129	-0.121	-0.010	-0.030	-.229**	-0.039	-.170*	0.052	0.164	0.134	-0.082	-0.117	0.048	-0.013
	p	0.385	0.865	0.467	0.134	0.159	0.906	0.725	0.007	0.649	0.048	0.547	0.056	0.120	0.344	0.175	0.575	0.885
**Acetic acid**	r	0.164	0.128	0.119	0.134	.175*	.229**	.291**	0.116	0.097	0.099	0.007	0.080	0.037	0.079	0.086	0.069	0.095
	p	0.057	0.136	0.168	0.120	0.041	0.007	0.001	0.178	0.264	0.252	0.934	0.352	0.667	0.360	0.317	0.427	0.269
**Betaine**	r	0.151	0.051	.183*	0.066	.181*	0.139	0.091	0.053	0.035	0.126	-0.009	0.015	0.023	0.053	0.070	-0.083	-0.108
	p	0.079	0.556	0.033	0.445	0.035	0.105	0.292	0.540	0.688	0.143	0.920	0.859	0.792	0.538	0.418	0.335	0.212
**Acetoacetate**	r	-0.049	-0.011	-0.017	0.073	-0.015	0.030	0.168	-0.037	-0.022	0.023	0.141	0.065	0.066	-0.027	0.059	-0.039	-0.086
	p	0.575	0.903	0.841	0.400	0.860	0.729	0.050	0.672	0.796	0.791	0.101	0.450	0.444	0.752	0.492	0.656	0.320
**Carnitine**	r	-0.044	-0.120	0.075	-0.011	-0.023	0.095	-0.011	-0.101	-0.107	-0.072	0.102	0.146	0.036	-0.088	0.062	0.003	0.003
	p	0.612	0.165	0.382	0.900	0.788	0.273	0.896	0.240	0.216	0.404	0.239	0.091	0.680	0.307	0.473	0.977	0.976
**Creatine**	r	0.035	0.048	0.010	-0.014	0.037	0.040	0.063	-0.082	-0.004	-0.023	0.079	0.074	0.098	-0.014	-0.090	0.010	-0.049
	p	0.688	0.577	0.907	0.869	0.667	0.646	0.468	0.345	0.963	0.791	0.358	0.392	0.255	0.869	0.297	0.912	0.568

Commented [SM2]: With this table it is more difficult because font 9 is not enough to avoid changing the table format. I have reduced it to font size 6 keeping the same Theme font: Palatino Linotype

Commented [SM3]:

**Dimethylamine**	r	-0.094	-0.166	0.052	-0.053	-0.068	0.055	-0.066	-0.142	-0.079	-0.056	0.056	0.104	-0.053	0.081	0.121	0.134	0.060
	p	0.275	0.053	0.550	0.536	0.432	0.523	0.448	0.099	0.363	0.519	0.514	0.228	0.540	0.347	0.159	0.121	0.487
**Dimethylglycine**	r	-0.160	-0.078	-.182*	0.008	-.199*	0.009	0.004	-0.108	0.003	-0.040	0.051	-0.033	-0.145	-.188*	-0.071	-0.045	-0.091
	p	0.062	0.365	0.034	0.925	0.020	0.916	0.967	0.212	0.976	0.641	0.559	0.703	0.091	0.029	0.411	0.605	0.293
**Citrate**	r	.208*	0.153	.174*	0.019	.190*	0.113	0.109	0.072	0.056	0.135	-0.061	-0.023	0.043	0.029	0.020	-.186*	-.189*
	p	0.015	0.075	0.043	0.828	0.027	0.190	0.206	0.402	0.517	0.117	0.483	0.790	0.623	0.736	0.819	0.030	0.027
**Choline**	r	0.013	-0.071	0.130	0.021	0.019	0.080	0.075	-0.080	-0.064	-0.051	0.149	.258**	0.095	0.000	-0.037	0.052	-0.016
	p	0.876	0.412	0.132	0.812	0.824	0.354	0.388	0.356	0.461	0.552	0.083	0.002	0.270	0.996	0.666	0.551	0.853
**Ethanol**	r	-0.010	0.051	-0.080	-0.034	0.008	-0.014	0.114	0.049	-0.055	0.034	0.039	0.011	0.128	-0.055	-0.021	-0.091	-0.145
	p	0.907	0.556	0.356	0.694	0.924	0.874	0.188	0.572	0.524	0.695	0.649	0.899	0.137	0.522	0.809	0.294	0.091
**L- Proline**	r	0.010	-0.055	0.077	-0.009	0.039	0.121	-0.014	0.002	-0.008	0.056	0.107	0.041	-0.080	0.042	-0.060	-0.016	-0.077
	p	0.906	0.525	0.372	0.916	0.652	0.160	0.867	0.979	0.930	0.517	0.213	0.634	0.356	0.629	0.487	0.857	0.374
**L-Threonine**	r	-0.049	-0.059	-0.004	-0.093	-0.078	-0.112	0.001	-0.062	-0.048	0.003	-0.162	-0.077	0.063	-0.106	0.035	0.057	0.102
	p	0.570	0.493	0.965	0.280	0.370	0.196	0.993	0.476	0.582	0.972	0.060	0.373	0.468	0.220	0.683	0.507	0.235
**L-Asparagine**	r	0.038	-0.018	0.123	0.036	0.069	0.045	0.034	0.042	-0.037	0.078	-0.005	0.115	0.025	-0.023	-0.029	-0.113	-0.087
	p	0.663	0.831	0.155	0.680	0.422	0.605	0.693	0.627	0.671	0.370	0.953	0.181	0.772	0.793	0.736	0.189	0.313
**D-Glucose**	r	0.100	0.013	0.165	-0.057	0.091	0.033	0.059	-0.006	-0.063	-0.107	0.162	0.047	0.063	0.043	-0.113	0.021	-0.053
	p	0.247	0.881	0.054	0.513	0.293	0.703	0.494	0.949	0.467	0.216	0.059	0.589	0.469	0.622	0.190	0.809	0.537
**Glycine**	r	-0.071	-0.163	0.091	0.058	-0.034	0.023	-0.007	-0.080	.210*	0.003	-0.033	-0.070	-.238**	-0.063	.178*	-0.046	-0.073
	p	0.415	0.057	0.290	0.501	0.699	0.794	0.934	0.352	0.014	0.977	0.706	0.419	0.005	0.464	0.039	0.592	0.396
**Glycerol**	r	-0.086	-.187*	0.123	0.021	-0.055	0.061	-0.028	-0.120	.175*	0.011	-0.021	-0.028	-.220*	-0.047	.185*	-0.013	-0.036
	p	0.320	0.030	0.153	0.806	0.523	0.477	0.745	0.162	0.041	0.894	0.810	0.742	0.010	0.589	0.031	0.882	0.680
**Fumaric acid**	r	0.093	0.004	0.148	-0.134	0.051	0.047	0.034	-0.103	-0.001	0.031	-0.085	0.157	0.112	0.032	0.004	0.051	0.009
	p	0.282	0.964	0.085	0.121	0.554	0.585	0.690	0.233	0.994	0.719	0.326	0.068	0.195	0.712	0.963	0.555	0.919
**Formate**	r	0.009	-0.026	0.093	0.084	0.040	0.084	0.123	-0.083	0.049	0.005	0.035	0.057	0.065	0.013	-0.031	0.018	0.008
	p	0.914	0.767	0.282	0.332	0.645	0.329	0.155	0.339	0.574	0.952	0.683	0.513	0.455	0.883	0.723	0.839	0.927
**L-Glutamic acid**	r	-0.090	-0.150	0.014	0.040	-0.010	0.048	-0.065	-0.094	-.190*	-0.129	0.127	.190*	-0.089	0.038	-0.006	0.027	-0.060
	p	0.297	0.082	0.872	0.644	0.908	0.578	0.449	0.275	0.027	0.135	0.142	0.026	0.304	0.661	0.941	0.759	0.490
**Hypoxanthine**	r	0.045	0.055	0.039	0.115	0.086	-0.089	0.093	0.018	0.003	0.038	0.061	-0.081	0.001	-0.021	-0.084	0.106	0.153
	p	0.607	0.527	0.654	0.183	0.317	0.304	0.280	0.831	0.974	0.662	0.482	0.348	0.995	0.813	0.331	0.221	0.076
**Tyrosine**	r	0.102	0.081	0.104	0.084	0.147	0.113	0.001	0.104	0.064	0.073	0.063	0.069	0.072	.170*	0.005	-0.110	-0.138
	p	0.237	0.348	0.228	0.332	0.087	0.191	0.993	0.230	0.461	0.400	0.467	0.422	0.404	0.048	0.951	0.204	0.110
**L-Phenylalanine**	r	-0.032	-0.087	0.101	0.096	0.017	0.077	0.033	-.187*	-0.059	-0.078	0.102	0.149	0.060	0.012	-0.067	0.007	-0.029
	p	0.708	0.314	0.243	0.268	0.844	0.372	0.701	0.030	0.498	0.364	0.237	0.084	0.485	0.892	0.440	0.932	0.737
**LAlanine**	r	-0.013	-0.070	0.051	0.045	0.037	0.058	-0.032	0.089	-0.009	0.014	0.065	0.109	-0.060	0.070	-0.035	-0.022	-0.031
	p	0.876	0.418	0.555	0.605	0.670	0.503	0.710	0.305	0.914	0.869	0.454	0.208	0.488	0.418	0.682	0.797	0.718
**Mannose**	r	-.257**	-.254**	-0.103	-0.023	-.252**	-0.064	-0.079	-0.125	0.017	-0.023	0.098	-0.004	-0.140	-.209*	0.095	-0.133	-0.039
	p	0.002	0.003	0.234	0.793	0.003	0.462	0.358	0.146	0.840	0.789	0.255	0.964	0.103	0.015	0.272	0.121	0.649
**Isoleucine**	r	0.046	-0.043	0.127	0.016	0.077	-0.024	0.042	0.012	-0.149	-0.054	0.046	0.093	0.028	0.010	-0.162	0.006	-0.063
	p	0.596	0.618	0.140	0.853	0.372	0.781	0.626	0.890	0.084	0.532	0.592	0.283	0.749	0.904	0.060	0.944	0.469
**L-Histidine**	r	0.076	-0.056	0.155	-0.029	0.029	-0.016	-0.160	-0.029	-.203*	0.099	-0.048	0.042	0.038	-0.081	-0.087	-0.022	-0.088
	p	0.379	0.516	0.071	0.739	0.741	0.857	0.062	0.741	0.018	0.249	0.576	0.628	0.660	0.348	0.312	0.796	0.309
**L-Lysine**	r	0.014	-0.080	0.148	0.054	0.035	0.114	-0.020	-0.058	0.025	0.016	0.123	0.015	0.026	0.028	-0.027	0.026	0.016
	p	0.868	0.356	0.086	0.535	0.686	0.186	0.816	0.502	0.775	0.853	0.152	0.859	0.768	0.745	0.753	0.763	0.856
**L-Serine**	r	-.213*	-.184*	-0.075	-0.047	-.220**	-0.089	-0.083	-0.020	-0.126	-0.023	0.033	0.051	-0.053	-0.108	-0.017	0.016	0.095
	p	0.013	0.032	0.387	0.588	0.010	0.305	0.337	0.819	0.144	0.791	0.699	0.559	0.543	0.212	0.845	0.850	0.273
**L-Lactic acid**	r	-0.058	-0.150	0.098	0.012	-0.004	0.028	0.033	-0.131	-0.143	-0.149	0.103	.250**	0.003	0.065	-0.040	-0.050	-0.083
	p	0.503	0.080	0.256	0.888	0.960	0.745	0.702	0.129	0.096	0.083	0.235	0.003	0.973	0.452	0.640	0.566	0.338
**Aspartate**	r	-0.142	-.189*	0.045	-0.072	-0.121	0.035	-0.071	-.178*	-0.059	-0.004	-0.055	0.125	-0.019	-0.063	-0.072	0.055	0.047
	p	0.100	0.028	0.599	0.405	0.159	0.682	0.411	0.038	0.495	0.964	0.526	0.147	0.828	0.467	0.402	0.522	0.583
**Myo-inositol**	r	-0.122	-0.163	-0.016	-0.123	-0.161	0.021	-0.052	-.210*	-0.146	-0.140	0.100	.186*	0.005	-0.037	0.019	0.034	0.106
	p	0.157	0.059	0.854	0.153	0.061	0.810	0.547	0.014	0.091	0.105	0.249	0.030	0.956	0.671	0.826	0.695	0.220
**L-Ornithine**	r	0.004	-0.021	0.036	0.084	0.033	0.121	0.062	-0.061	0.088	0.063	-0.032	-0.049	0.060	0.075	-0.087	-0.054	-0.089

	p	0.964	0.810	0.682	0.329	0.706	0.162	0.471	0.479	0.306	0.465	0.710	0.572	0.484	0.388	0.314	0.529	0.306
**Pyruvate**	r	-.192*	-.222**	-0.049	-0.058	-0.152	0.025	-0.086	-.192*	-0.128	-0.30**	0.134	.224**	-0.111	-0.088	-0.093	-0.047	-0.156
	p	0.025	0.009	0.568	0.505	0.077	0.776	0.317	0.025	0.137	0.000	0.119	0.009	0.199	0.308	0.282	0.588	0.069
**Succinate**	r	0.039	0.005	0.076	0.138	0.104	0.046	0.134	-0.037	-0.036	0.095	0.038	0.090	0.036	0.099	0.073	-0.009	0.007
	p	0.650	0.953	0.379	0.110	0.228	0.592	0.121	0.669	0.676	0.270	0.661	0.299	0.680	0.252	0.399	0.913	0.932
**Pyroglutamic acid**	r	0.024	-0.049	0.104	0.126	0.096	0.045	0.105	0.005	0.007	0.126	-0.021	0.031	-0.023	0.132	0.116	-0.034	-0.025
	p	0.778	0.572	0.229	0.145	0.266	0.600	0.225	0.949	0.935	0.144	0.804	0.722	0.793	0.125	0.181	0.691	0.769
**Sarcosine**	r	-0.002	-0.079	0.134	-0.024	0.015	-0.041	-0.049	-0.063	0.054	0.052	-.208*	-0.006	0.010	-0.048	-0.110	0.095	0.134
	p	0.985	0.362	0.119	0.781	0.858	0.638	0.569	0.465	0.529	0.548	0.015	0.946	0.905	0.576	0.203	0.272	0.120
**Xanthine**	r	0.002	0.037	-0.082	0.095	0.053	-0.100	0.086	0.067	0.036	0.093	-0.121	-0.004	0.107	0.027	-0.066	0.058	0.098
	p	0.986	0.673	0.343	0.269	0.541	0.249	0.322	0.438	0.676	0.282	0.162	0.964	0.215	0.757	0.446	0.502	0.257
**Urea**	r	0.036	0.026	0.061	0.132	0.124	-0.019	0.039	0.030	-0.115	-0.026	-0.076	0.105	0.121	-0.145	-0.084	-0.105	-0.135
	p	0.678	0.763	0.481	0.126	0.151	0.823	0.652	0.733	0.181	0.761	0.378	0.222	0.162	0.091	0.333	0.224	0.116
**3-Hydroxybutyric acid**	r	-0.073	-0.057	0.024	-0.027	-0.067	0.060	0.168	-0.044	-0.001	0.027	0.106	0.074	0.004	-0.004	0.060	-0.034	-0.064
	p	0.397	0.509	0.778	0.758	0.439	0.489	0.051	0.614	0.990	0.758	0.221	0.390	0.961	0.965	0.489	0.694	0.456
**3-Methylhistidine**	r	-0.021	-.180*	.198*	0.029	-0.014	0.162	-0.029	-0.043	-0.030	-0.136	0.061	0.094	-0.086	0.059	0.124	0.022	-0.031
	p	0.811	0.036	0.021	0.740	0.875	0.060	0.737	0.621	0.732	0.115	0.482	0.274	0.318	0.495	0.151	0.797	0.720
**L-Arginine**	r	-0.067	-0.023	-0.076	0.166	0.000	-0.024	0.006	-0.051	-0.039	0.067	-0.020	0.022	-0.099	-0.074	-0.051	-0.059	0.007
	p	0.441	0.791	0.379	0.054	0.997	0.779	0.949	0.555	0.649	0.438	0.813	0.803	0.252	0.393	0.557	0.497	0.932
**Creatinine**	r	0.068	-0.031	0.139	0.028	0.114	0.100	-0.023	0.027	0.002	0.052	0.044	0.097	0.045	-0.005	-0.097	0.017	-0.081
	p	0.429	0.716	0.106	0.751	0.186	0.245	0.794	0.753	0.982	0.546	0.614	0.260	0.599	0.951	0.263	0.846	0.348
**L-Glutamine**	r	0.049	0.017	0.032	.303**	.175*	-0.089	0.122	-0.030	-0.051	0.012	-0.090	-0.035	0.014	-0.105	0.083	-.199*	-0.127
	p	0.574	0.848	0.715	0.000	0.041	0.302	0.157	0.731	0.553	0.889	0.299	0.683	0.868	0.225	0.335	0.020	0.139
**L-Leucine**	r	-0.013	-0.077	0.099	0.046	0.035	0.056	0.065	-0.096	-0.121	-0.056	0.061	0.128	0.063	0.002	-0.085	0.035	-0.005
	p	0.878	0.375	0.252	0.595	0.690	0.516	0.451	0.265	0.161	0.516	0.477	0.138	0.469	0.978	0.323	0.687	0.957
**Malonate**	r	0.089	-0.026	.185*	-0.109	0.058	0.132	0.023	0.068	0.003	0.133	0.019	0.054	0.056	0.069	-0.120	-0.043	-0.104
	p	0.305	0.763	0.031	0.208	0.499	0.126	0.789	0.429	0.972	0.122	0.822	0.531	0.515	0.422	0.165	0.623	0.229
**Methionine**	r	0.007	-0.050	0.112	0.133	0.057	0.104	0.118	-0.076	-0.013	-0.030	0.100	0.099	0.065	0.060	-0.034	-0.003	-0.023
	p	0.931	0.563	0.195	0.121	0.512	0.229	0.172	0.377	0.880	0.727	0.247	0.251	0.451	0.491	0.694	0.974	0.789
**Hippuric acid**	r	0.040	-0.021	0.077	-0.108	0.015	-0.063	0.077	0.004	-0.034	0.013	-0.121	0.109	.196*	-0.095	-0.137	0.092	0.107
	p	0.642	0.806	0.371	0.212	0.860	0.468	0.371	0.960	0.697	0.881	0.162	0.208	0.022	0.274	0.113	0.288	0.213
**Isovaleric acid**	r	-0.096	-.173*	0.072	0.048	-0.062	-0.085	0.010	-0.104	-0.166	-0.086	-0.053	0.017	-0.152	-0.084	-0.075	-0.039	-0.001
	p	0.265	0.044	0.402	0.582	0.475	0.327	0.906	0.226	0.053	0.317	0.542	0.841	0.077	0.328	0.384	0.654	0.986
**3-Hydroxyisovalericacid**	r	0.008	0.002	0.038	0.035	0.011	-.196*	-0.063	-0.020	0.016	0.099	-.270**	-0.123	0.132	-0.155	-0.077	0.059	0.078
	p	0.930	0.986	0.659	0.689	0.900	0.022	0.470	0.817	0.849	0.252	0.002	0.155	0.125	0.072	0.373	0.495	0.364
**Isopropyl alcohol**	r	-0.111	-0.029	-0.098	-0.062	-0.158	-0.002	0.075	-0.055	0.002	-0.066	0.113	.175*	0.091	-0.052	0.073	-0.026	-0.032
	p	0.199	0.734	0.254	0.470	0.066	0.982	0.383	0.528	0.979	0.445	0.189	0.041	0.291	0.546	0.397	0.763	0.715
**Valine**	r	0.083	-0.020	.197*	0.020	0.106	0.125	-0.019	-0.011	-0.002	0.000	0.081	0.094	0.008	0.088	-0.044	-0.028	-0.047
	p	0.340	0.817	0.022	0.813	0.219	0.147	0.830	0.901	0.982	0.998	0.350	0.279	0.927	0.306	0.609	0.749	0.585
**Tryptophan**	r	-0.135	-0.051	-0.078	0.079	-0.098	-0.160	0.154	-0.140	-0.098	-0.002	0.012	0.011	0.060	-0.106	-0.054	0.083	0.060
	p	0.117	0.557	0.364	0.360	0.255	0.063	0.073	0.104	0.254	0.982	0.886	0.896	0.487	0.218	0.532	0.340	0.486
**Acetone**	r	0.017	-0.044	0.161	0.011	0.035	0.111	0.153	-0.094	0.099	0.083	0.103	0.077	0.077	0.061	0.109	-0.024	-0.051
	p	0.845	0.611	0.062	0.895	0.689	0.199	0.075	0.276	0.252	0.339	0.234	0.372	0.375	0.478	0.204	0.785	0.556
**Isobutyric acid**	r	0.006	0.004	0.042	0.088	0.070	-0.082	0.091	0.019	-0.050	-0.097	0.016	0.163	0.103	-0.110	-0.088	-.181*	-.213*
	p	0.949	0.966	0.626	0.310	0.417	0.346	0.292	0.827	0.561	0.263	0.849	0.058	0.231	0.203	0.307	0.035	0.013
**Methanol**	r	0.040	0.062	-0.027	0.076	0.054	0.110	0.139	-0.134	0.011	-0.042	0.147	0.037	0.071	-0.067	-0.064	0.002	-0.090
	p	0.647	0.471	0.756	0.377	0.532	0.203	0.107	0.120	0.896	0.631	0.087	0.668	0.412	0.435	0.462	0.983	0.298
**Propyleneglycol**	r	0.060	0.060	0.083	0.091	0.089	0.001	0.079	-0.045	0.005	-0.056	-0.002	0.121	-0.002	-0.116	-0.029	0.006	-0.008
	p	0.486	0.487	0.339	0.294	0.301	0.993	0.364	0.601	0.954	0.518	0.980	0.159	0.982	0.178	0.740	0.947	0.929
**Dimethylsulfone**	r	-0.081	-0.113	0.015	.193*	0.010	-0.059	0.060	0.023	0.050	0.017	0.050	-0.069	0.015	-0.105	0.079	-0.050	-0.063
	p	0.349	0.192	0.866	0.024	0.905	0.496	0.485	0.793	0.563	0.848	0.562	0.425	0.859	0.224	0.363	0.561	0.468

Table S7. 14-point criteria used to measure adherence to MD on the MEDDINI study.14 POINT MEDITERRANEAN DIET SCORE (MDS).

Questions.	Criteria for 1 point
1. Do you use olive oil or rapeseed oil as your main cooking fat?	Yes
2. Do you use olive or rapeseed oil-based spreads (e.g. Golden olive, Olivio, Bertolli)?	Yes
3. How much olive/ rapeseed oil do you consume in a given day? Including oil used for frying, salads, out-of-house meals etc (in tablespoons)<br>How much olive or rapeseed oil-based spread do you consume in a given day? (in teaspoons)	≥ 4 tbsp oil / day<br>and/or<br>3 tsp spread/ day
4. How many portions of fruit (including natural fruit juices) do you consume per day? (1 portion = 1 apple/banana (80g), small glass juice (150ml)	≥ 2 portions/ day
5. How many vegetable servings do you consume per day? Including raw/ cooked vegetables, salad but not including potatoes (1 serving: 3 tablespoons/80g)	≥ 3 portions/ day
6. How many servings of legumes (peas, beans and lentils including kidney beans, baked beans, chickpeas, red lentils etc) do you consume per week? (1 serving :3 tablespoons/ 80g)	≥ 3 servings/ week
7. How many servings of red meat including beef, pork, lamb and minced beef do you consume per week? (1 serving: medium portion/ 100–150 g)	≤ 2 servings /week
8. How many servings of processed meat including ham, bacon, sausages, meat pies and other meat products etc.) do you consume per week? (1 serving: medium portion/ 100–150 g)	≤ 1 serving /week
9. How many servings of chicken/ turkey do you consume per week? (1 serving: medium portion/ 100-150g)	2 servings /week
10. How many servings of fish (tuna, cod, haddock, salmon, mackerel, herring, and sardines etc, including tinned varieties, excluding crumbed or battered fish) or shellfish do you consume per week? (1 serving: 1 fillet/small fish or 140g)	≥ 3 servings /week
11. Do you preferentially consume wholegrain bread and/ or cereal and/ or rice and/ or pasta instead of non-wholegrain (white) varieties?	Yes
12. How many servings of natural nuts do you consume per week? (1 serving: 1 small handful/ 30 g)	≥ 3 servings /week
13. How many times per week do you consume sweet foods (including biscuits, buns, pastries, chocolate, sweets, sweet or carbonated drinks or desserts)?	≤ 3 times/ week
14. How often would you consume up to 3 small glasses of wine or equivalent other alcoholic beverages per week? (1 small glass:125ml)	1–3 glasses or equivalent ≥ 3 days/ week
